# A novel mutation of the *Keratin 12* gene responsible for a severe phenotype of Meesmann's corneal dystrophy

**Published:** 2007-06-21

**Authors:** Lori S. Sullivan, Eric B. Baylin, Ramon Font, Stephen P. Daiger, Jay S. Pepose, Thomas E. Clinch, Hisashi Nakamura, Xinping C. Zhao, Richard W. Yee

**Affiliations:** 1 Hermann Eye Center, University of Texas Health Science Center at Houston, Houston, TX; 2 Department of Ophthalmology and Visual Science, the University of Texas Health Science Center at Houston, Houston, TX; 3 School of Public Health, The University of Texas Health Science Center at Houston, Houston, TX; 4 The Cullen Eye Institute, Baylor College of Medicine, Houston, TX; 5 Department of Ophthalmology and Visual Sciences, Washington University Medical Center, St. Louis, MO; 6 John A. Moran Eye Center, University of Utah Health Sciences Center, Salt Lake City, UT

## Abstract

**Purpose:**

To determine if a mutation within the coding region of the *keratin 12* gene (*KRT12*) is responsible for a severe form of Meesmann's corneal dystrophy.

**Methods:**

A family with clinically identified Meesmann's corneal dystrophy was recruited and studied. Electron microscopy was performed on scrapings of corneal epithelial cells from the proband. Mutations in the *KRT12* gene were sought using direct genomic sequencing of leukocyte DNA from two affected and two unaffected family members. Subsequently, the observed mutation was screened in all available family members using polymerase chain reaction and direct sequencing.

**Results:**

A heterozygous missense mutation (Arg430Pro) was found in exon 6 of *KRT12* in all 14 affected individuals studied. Unaffected family members and 100 normal controls were negative for this mutation.

**Conclusions:**

We have identified a novel mutation in the *KRT12* gene that is associated with a symptomatic phenotype of Meesmann's corneal dystrophy. This mutation results in a substitution of proline for arginine in the helix termination motif that may disrupt the normal helix, leading to a dramatic structural change of the keratin 12 protein.

## Introduction

Meesmann's corneal dystrophy (MCD), a bilaterally symmetric disorder, is characterized by fragility of the corneal epithelium [1,2]. MCD exhibits myriads of fine punctate opacities and cysts in the epithelium and occasionally in Bowman membrane and can be diagnosed on slit-lamp examination. While early disease is often asymptomatic, multiplication of the cysts throughout late adolescence and adulthood can cause epithelial erosion, leading to lacrimation, photophobia, and pain. In most cases, visual acuity is rarely affected; however, subepithelial scarring can occasionally produce irregular astigmatism and irrevocable visual deficits. Light microscopy often shows a thickened basement membrane and electron microscopy will reveal affected corneal epithelial cells with highly characteristic intracytoplasmic cysts containing an electron dense material referred to as "peculiar" substance [2,3].

The structural integrity of corneal epithelial cells derives largely from its composition of cytoskeletal intermediate filaments, specifically keratin 3 and keratin 12 [4,5]. These molecules function as heterodimers of type I and type II keratin [6], whereby disruption of a single keratin polymer can compromise epithelial structure and function [7]. Until the present study, 14 mutations in *KRT12* and two mutations in *KRT3* had been found in MCD patients. All mutations were located in either helix-initiation or helix-termination motif [8-17]. The motifs were highly conserved among keratin proteins, and the domains are important in intermediate filament assembly and are likely involved in mediating end-to-end interactions between keratin heterodimers. Interestingly, there were more mutations reported in the helix-initiation motif than in the helix-termination motif of these two keratin genes. For example, 10 different mutations have been identified thus far in the helix-initiation motif of KRT12, including substitution of amino acid M129T [8], Q130P [9], N133K [10], R135S [11], R135G [12], R135I [12], R135T [13], A137P [14], L140R [12], and V143L [13]. However, there have been only four mutations reported in the helix-termination motif in KRT12: I426V [15], I426S [16], Y429D [12], and Y429C [17]. Also, the two reported mutations in KRT3 (R503P [17], E509K [13]) are in the helix-termination motif and not the helix-initiation motif.

In this paper, we report a new case of MCD found in a family who exhibited severe spectrum of disease. We performed mutation screening on the family and discovered a novel missense mutation in the helix-termination motif of KRT12.

## Methods

### Subjects

This study was approved by the institution review board of the University of Texas Health Science Center at Houston. All patients were informed about the study and signed a consent form in accordance with guidelines set forth by the Declaration of Helsinki. A large family with 17 members affected with Meesmann's corneal dystrophy was identified and given complete eye exams. For detailed examination of patient corneas, bilateral corneal scrapings were performed on the proband, and scrapings of corneal epithelial cells were subsequently fixed in 2% glutaraldehyde and visualized by using conventional electron microscopy.

### Detection of *KRT12* mutations

**Figure 1:**
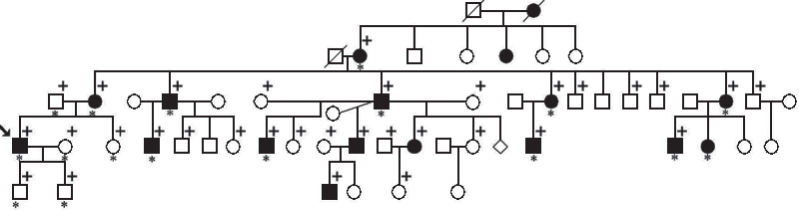
Pedigree of a large family with Meesmann's corneal dystrophy showing a typical autosomal dominant mode of inheritance. Squares represent males and circles represent females. Filled symbols mark affected individuals. Individuals who contributed DNA samples and were tested for the R430P mutation are marked with a plus sign. Asterisks denote participants who were clinically examined by the authors. Arrow indicates the proband.

Genomic DNA was extracted from peripheral blood obtained from all family members (Figure 1). Mutation screening of the *KRT12* gene was done initially in one affected and one unaffected family member. The entire coding region (all eight exons) was examined by direct polymerase chain reaction (PCR) sequencing. The primers used for amplification of all exons of *KRT12* are shown in Table 1. PCR products were examined on 2% agarose gels and then sequenced using an AB 3100 genetic analyzer (Applied Biosystems, Foster City, CA). A mutation in exon 6 was confirmed by using the restriction enzyme *Fau*I (New England Biolabs, Beverly, MA), according to the manufacturer's protocol, to digest PCR products from the proband and his unaffected sister. Digested samples were run on 2.5% agarose gels, and the bands were visualized by ethidium bromide staining. Also, DNA samples from the remaining family members were PCR amplified and sequenced for exon 6 of *KRT12*. The mutation was further verified by sequencing of PCR products amplified from 100 Centre d'Etude du Polymorphisme Humain (CEPH) control samples.

**Table 1:** Primers for polymerase chain reaction direct sequencing.

**Exon**	**5' primer**	**3' primer**	**PCR product size**
1-1	GAGTGAAACAACCTGGGATGAGAA	GAGGGCTGGGGATGGGATTTG	429
1-2	TGCAGTCCCAGTTCCTCGTGTTTC	ACTGTCCCGGCGGCTCTCCTC	460
1-3	CCCCTGGGTCTGCCTATCACACTC	TGGGAATACAGGCAACAGACTAAT	430
2	CAATGAAGGCAGGACAGTAGGA	TAGTCI I I IAGG GCTTCAATCTT	201
3	ATGGCCAI I I IAACAGGGAGACA	TCATCGCTGGTAGGAAAGTATTG	534
4	CATGGCGAGCTGGGACTGTAG	GTCCTCTTGGGCCCCTTCCTA	470
5	TCTGCACGTGGGAGGGAAATG	GCGGGCGCAGTATGAAACCA	427
6	AACCCCATTCCTTCTATTTCTGCT	CCCCCTGGCTGTCTTTGCTGTT	591
7	CCTCAAGCGATCCTCCCACCTC	AGCCACCTGAACCACCTACTCTAA	501
8	TCCGGGTTACCAGAAGAAAGT	GAAGCCTACATTAAACAACCAGTG	460

Three different PCR products were used for sequence analysis of exon 1 since it was so large.

## Results

### Clinical Exam

**Figure 2:**
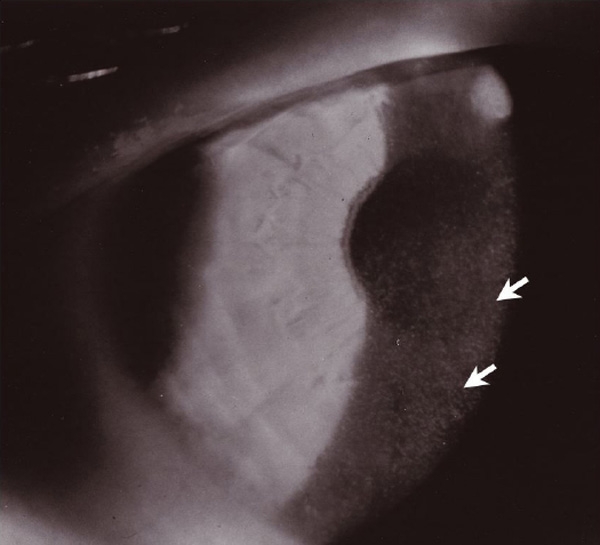
Slit-lamp photograph demonstrating discrete microcysts in the anterior corneal epithelium. Microcysts ranged from clear vesicles to opacified inclusions.

The proband was a 33-year-old male with symptomatic MCD from birth. He presented with symptoms of photophobia, lacrimation, periodic burning, and irritation. He had flare-ups almost monthly that lasted from two days to one week, followed by remission. Unlike most patients with MCD, his visual acuity was significantly impaired (20/200 visual acuity score), and he wore corrective lenses. Slit-lamp examination disclosed dense microcysts in the visual axis limited to the anterior epithelium in addition to significant anterior basement membrane scarring (Figure 2). Clinical histories obtained from family members were remarkably similar. The general impression by collective slit-lamp examinations was consistent with MCD. In this family, however, MCD presents an unusually severe spectrum of disease.

### Electron Microscopy

**Figure 3:**
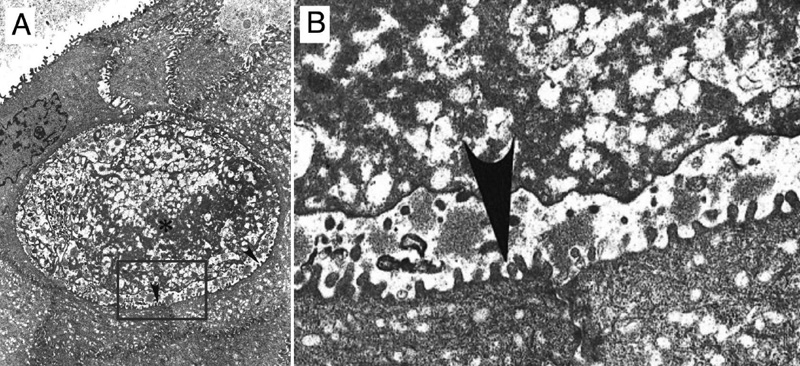
Presence of a "peculiar" electron-dense substance in the intraepithelial cysts of the corneal epithelium sampled from the proband. **A**: Electron micrograph of corneal epithelium depicting an intraepithelial cyst containing a "peculiar" electron-dense substance (asterisk) intermixed with small vacuoles and electron-dense filamentous material (original magnification 5,400x). **B**: Higher magnification of **A**. The cyst was bordered by numerous microvillous processes (arrowheads).

Electron microscopy analysis of the proband showed intraepithelial cysts involving the superficial cell layers of the cornea. The cysts were bordered by microvillous processes, indicating they were formed by acantholysis (Figure 3). The cysts contained a "peculiar" substance intermixed with numerous round vacuoles that were surrounding electron-dense elongated filamentous material [10].

### Molecular Genetic Analysis

**Figure 4:**
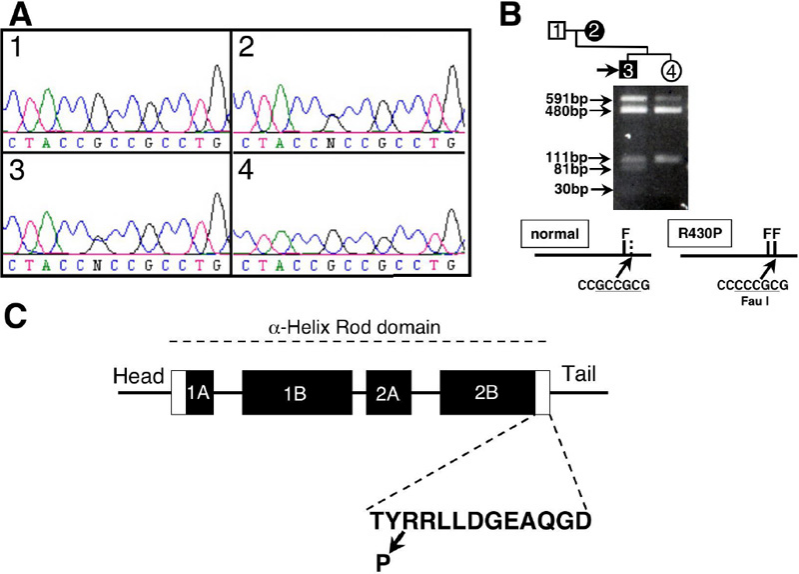
Mutation analysis of the *KRT12* gene. **A**: Bidirectional sequence analysis of the *KRT12* gene. The results shown are in the sense direction. *KRT12* sequence with in the helix-terminal motif of rod domain 2B in affected family members (2 and 3) showing a G to C transversion at the at the 2nd position of codon 430 that results in an amino acid change from arginine to proline. 1, unaffected father; 2, affected mother; 3, proband and 4, unaffected sister. **B**: Restriction endonuclease analysis was used to detect R430P mutation. Amplicons of exon 6 were digested with *Fau*I, size fractionated on a 2.5% agarose gel, and visualized under ultraviolet light after staining with ethidium bromide. An additional *Fau*I site (generated by the R430P mutation) converts the 111 bp fragment into 81 and 30 bp fragments. Due to poor enzyme activity, undigested PCR product (591 bp) was observed. The 30 bp fragment was difficult to visualize. **C**: The domain structure of KRT12 and the mutation position found in MCD family in this study is shown. The rod domain comprised four segments (1A, 1B, 2A, and 2B), represented by filled boxes. The helix-initiation and -termination motif are represented by white boxes. The amino acid sequence for the helix-termination motif is shown.

Examination of phenotypes within this five-generation family indicated an autosomal dominant mode of inheritance (Figure 1). Since a majority of MCD patients have mutations in *KRT12*, we hypothesized that a mutation in *KRT12* might be associated with the phenotype observed in this family. To test this hypothesis, we sequenced the entire coding region of *KRT12* in selected family members. PCR analysis and direct DNA sequencing were performed in a screening panel of two affected and two unaffected individuals to determine if there was a mutation within the coding region of the *KRT12* gene that is associated with the form of MCD found in this family. This analysis identified a novel heterozygous missense mutation (Arg430Pro; CGC>CCC) in exon 6 of the *KRT12* gene in the affected individuals but not in the unaffected normal controls of the family (Figure 4A). Substitution of C for G in codon 430 resulted in the gain of a recognition site for the restriction enzyme *Fau*I. Since there is another *Fau*I site 30 bp apart from the site, digestion of the exon 6 PCR products from patients with this mutation produced bands of 220, 81, and 30 bp, while normal alleles produced bands of 220 and 111 bp (Figure 4B). As expected, *Fau*I digestion of PCR products generated a 81 bp fragment from the proband but not from his unaffected sister. Therefore, substitution of C for G in codon 430 is likely associated with MCD in this family. To determine whether this mutation was cosegregated with the disease, we examined and compared the sequence of exon 6 in all DNA samples obtained from the remaining family members. Sequence analysis showed that the affected individuals had this mutation but the unaffected individuals did not (data not shown). To further determine if this nucleotide substitution were associated with the disease phenotype, we performed sequence analysis of exon 6 of *KRT12* on the 100 CEPH control individuals. Our goal was to rule out the possibility that this alternation in DNA was a rare single nucleotide polymorphism occurring in the control population. Direct sequencing of exon 6 of the *KRT12* gene from these additional individuals failed to find the same sequence variation. Therefore, the Arg430Pro mutation that was identified and was cosegregated with the MCD in this family may be responsible for MCD of this family.

## Discussion

The family members we examined exhibited an unusually severe phenotype for MCD characterized by early onset of the disease, ocular irritation, and poor visual acuity. As an example, the proband has undergone four phototherapeutic keratectomy procedures for alleviation of recurrent symptoms. Irregular astigmatism has been problematic despite the improvement of the recurrent corneal erosions. This rather atypical presentation may reflect the unique Arg430Pro missense mutation, which lies within the helix termination motif of the α-helical rod domain of KRT12 (Figure 4B). The helix-termination motif is highly conserved among keratin proteins and is critical for keratin fiber formation. In addition to the helix initiation motif, mutations in this helix-termination motif are known to result in more severe phenotypes of other inherited epidermal diseases [18-22]. It is noteworthy that most documented keratin mutations responsible for MCD are in the helix initiation motif [9-12,14], and a few families have been identified with a mutation in the helix termination region [11,13,15,16]. It is possible that the clinical severity observed in our pedigree is due to the structural change of the keratin 12 protein caused by the replacement of arginine with a proline residue, which is particularly incompatible with an α-helical structure. The amino group of proline imposes both geometric and electrostatic constraints on the α-helix by elimination of N-C_α_ bond rotation and available hydrogen bonds. In contrast, a recent article described an essentially asymptomatic family with clinically diagnosed MCD caused by a different *KRT12* mutation. In this case, the identified *KRT12* mutation resulted from a substitution of a nonpolar for another nonpolar amino acid, probably causing a less severe disruption of the keratin protein structure (and a less severe phenotype) [15].

Unlike other mutations, the Arg430Pro leads to a replacement of a highly charged amino acid with a hydrophobic amino acid. We believe that the helix-termination motif in the mutant keratin protein may be no longer functional for the fiber formation. We are unaware of any other reports of *KRT12* mutations in MCD with the added instability of a proline substitution in the helix terminal motif. Our search of Medline, the Human Gene Mutation Database, or OMIM turned up no such mutation, although one group reported a proline substitution in the helix terminal motif in KRT3 [17]. We postulate that the severity of MCD in our pedigree is related specifically to the biochemical significance of the novel Arg430Pro mutation described herein.
